# Isochlorogenic Acid A Attenuates the Progression of Liver Fibrosis Through Regulating HMGB1/TLR4/NF-κB Signaling Pathway

**DOI:** 10.3389/fphar.2020.00582

**Published:** 2020-05-01

**Authors:** Xin Liu, Kai Huang, Ru Jiao Zhang, Dan Mei, Bo Zhang

**Affiliations:** ^1^Department of Pharmacy, Peking Union Medical College Hospital, Chinese Academy of Medical Sciences and Peking Union Medical College, Beijing, China; ^2^Key Laboratory of Molecular Pharmacology and Drug Evaluation (Yantai University), Ministry of Education, Yantai University, Yantai, China; ^3^Drug Clinical Trial Institution, Wuxi People’s Hospital, Nanjing Medical University, Wuxi, China; ^4^Health Science Center, Hebei University, Baoding, China

**Keywords:** isochlorogenic acid A, liver fibrosis, high-mobility group box 1, toll like receptor 4, nuclear factor-κB

## Abstract

Liver fibrosis, a chronic damage process related to further progression of hepatic cirrhosis, has yet no truly effective treatment. Isochlorogenic acid A (ICQA), isolated from a traditional Chinese herbal medicine named *Laggera alata* (DC.) Sch.Bip. ex Oliv. (Asteraceae), is proved to exhibit anti-inflammatory, hepatoprotective and antiviral properties. However, the actions of ICQA on liver fibrosis are poorly understood. The purpose of this study was to evaluate the actions of ICQA on liver fibrosis and clarify the underlying mechanism. It was found that ICQA had significant protective actions on liver injury, inflammation as we as fibrosis in rats. Meanwhile, ICQA prevented hepatic stellate cells (HSC) activation, indicated by its inhibitory effect on the overexpression of α-smooth muscle actin (α-SMA). In addition, the reduced fibrosis was found to be associated with the decreased protein expression of high-mobility group box 1 (HMGB1) as well as toll like receptor (TLR) 4. Simultaneously, ICQA can suppress the cytoplasmic translocation of HMGB1 in rat liver. Further investigations indicated that ICQA treatment dramatically attenuated the nuclear translocation of the nuclear factor-kB (NF-κB) p65 and suppressed the hepatic expression of p−IκBα in rats with liver fibrosis. Taken together, our study indicated that ICQA could protect against CCl_4_-induced liver fibrosis probably through suppressing the HMGB1/TLR4/NF-κB signaling pathways.

## Introduction

Liver fibrosis, characterized by the pathological synthesis and exposition of extracellular matrix (ECM), has been recognized as a key player in progression of hepatic cirrhosis and carcinoma (Atta, 2015; Sun and Kisseleva, 2015). It can be caused by various chronic liver injuries including alcoholic, non-alcoholic, drug-related, viral hepatitis, or autoimmune diseases (Bataller and Brenner, 2005). Although the mechanisms of the pathogenesis of liver fibrosis have been widely investigated over the past few decades, no effective treatment is available currently (Mallat and Lotersztajn, 2013; Schuppan and Kim, 2013).

Hepatic stellate cells (HSCs) play an important role in the pathogenesis of liver fibrosis. In normal condition, they keep quiescent in the perisinusoidal space, and can be activated following fibrogenic stimuluses (Iredale et al., 2013). Once activated, HSCs secrete inflammatory factors and fibrogenic cytokines, which can in turn promote the apoptosis of HSCs (Son et al., 2009; Mederacke et al., 2013; Seki and Schwabe, 2015). Hence, inhibition of the release of fibrogenic cytokines as well as inflammatory factors is thought to be potential approaches for the therapy of liver fibrosis (Seki and Schwabe, 2015; Zhang et al., 2019).

High mobility group box 1 protein (HMGB1), a highly conserved DNA binding nuclear protein, possesses the function of regulating gene transcription (Seki and Schwabe, 2015). Besides its nuclear roles, HMGB1 can also act as an inflammatory cytokine in pathological status and is widely considered as damage-associated molecular patterns (DAMPs) in recent years (Sims et al., 2010). Even though HMGB1 is recognized as a nuclear protein, it can be observed in cytoplasm of the necrotic cells. In the condition of cell damage, it can also be passively secreted into the extracellular space (Scaffidi et al., 2002). After release, extracellular HMGB1 binds to the cell surface receptors such as toll like receptor (TLR) 2, TLR4 (Yu et al., 2006). These pathways then trigger a signal cascade through activating the nuclear factor-κB (NF-κB), which causes further translocation and release of HMGB1 (Mencin et al., 2009). After the activation of NF-κB, cells secrete large numbers of inflammatory cytokines and initiate a cascade amplification of inflammatory responses, which play vital roles in the progress of liver fibrogenesis. Emerging evidences suggested that HMGB1/TLR4/NF-κB signaling was firmly associated with the activation of HSCs as well as the synthesis of ECM, and inhibition of HMGB1 could significantly reduce inflammatory response and ameliorate liver fibrosis (Luedde and Schwabe, 2011; Li et al., 2014). Hence, HMGB1 might be a critical regulator in liver fibrosis and inhibition of HMGB1 might be an effective approach for anti-inflammatory therapy of liver fibrosis.

**Figure 1:**
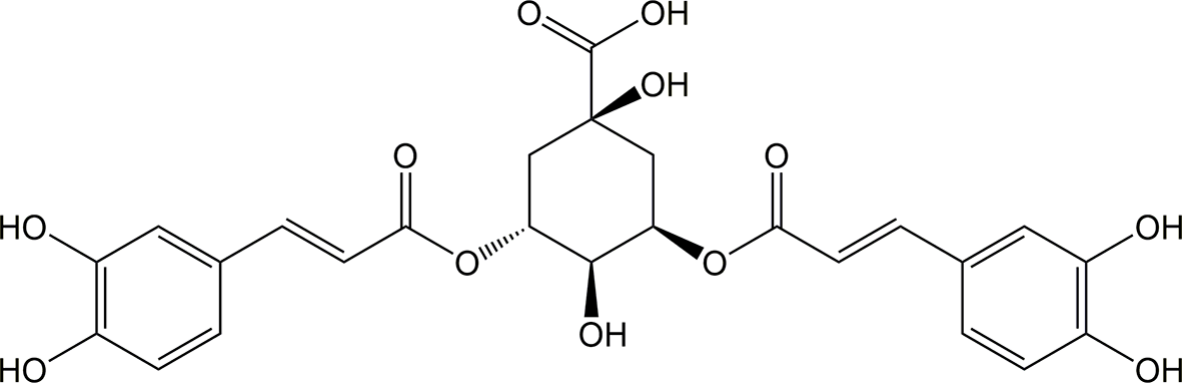
Chemical structure of ICQA.

Isochlorogenic acid A (ICQA, C_25_H_24_O_12_, molecular mass:516.45, CAS: 2450-53-5, **Figure 1**), naturally isolated from *Laggera alata* (DC.) Sch.Bip. ex Oliv. (*Asteraceae*), distributes primarily in tropical Southeast Asia and Africa. *L. alata* is a traditional Chinese medicine, particularly for the therapy of diseases related with inflammation (Zheng et al., 2003; Wu et al., 2006). Previous pharmacological investigations indicated that ICQA possesses significantly antiviral, neuroprotective and antioxidant properties (Ooi et al., 2006; Wu et al., 2007; Kim et al., 2012). In addition, ICQA showed significant hepatoprotective and anti-hepatitis B properties through inhibiting oxidation, which makes it to be a promising drug candidate for hepatitis (Hao et al., 2012). However, there is no specific evidence illustrating whether ICQA has protective effect on liver fibrosis. Therefore, the purpose of the present investigation was to observe the protective actions of ICQA on liver fibrosis and clarify the related mechanism.

## Materials and Methods

### Chemicals and Reagents

ICQA (purity >98%) was the product of Nanjing Jingzhu Bio-Technology Co. Ltd **(Nanjing, China)**. Carbon tetrachloride (CCl_4_) was the product of Shanghai Jinghua Scientific & Technological Research Institute (Shanghai, China). Antibodies against HMGB1, NF-κB p65 and β-actin were bought from Abcam (Cambridge, UK), and all the other antibodies were provided by Cell Signaling Technology (Beverly, MA, USA). Enzyme linked immunosorbent assay (ELISA) kits for rat TNF-α, IL-6, IL-1β were the products of R&D Systems (Minnesota, USA). Real-time PCR master mix was provided by Roche (Indianapolis, IN, USA) and ABI TaqMan primers/probes were obtained from Applied Biosystems (Foster City, CA, USA).

### Animals and Experimental Designs

Male Sprague–Dawley rats, 8–10 weeks old (240 ± 20 g, certificate no. SCXK2012-0001), were provided by the Beijing Vital River Experimental Animal Co., Ltd. (Beijing, China). All rats were maintained at a stable ambient temperature (23–25°C) with free access to food and water. All animal experimental procedures were approved by the Institutional Animal Care and Use Committee of the Peking Union Medical Hospital, Chinese Academy of Medical Sciences and Peking Union Medical College.

After acclimatization for 1 week, the rats were divided randomly into six groups (n = 10 per group) including control group, ICQA control group, model group and ICQA (10, 20, 40 mg/kg) treated group. Rats in the model and ICQA treated group were injected subcutaneously with 3 ml/kg CCl_4_ dissolved in olive oil (40%, V/V)) twice a week for 8 weeks. Meanwhile, animals in the ICQA treated group were simultaneously orally received different doses of ICQA (10, 20 or 40 mg/kg) dissolved in normal saline daily for 8 weeks. Animals in the ICQA control group were injected with the same volume of olive oil accompanied with orally given ICQA (40 mg/kg), while animals in the control group were administered with olive and normal saline. After 8 weeks of treatment, rats were sacrificed. Blood were collected, and serum was isolated from blood after centrifugation (1,200*g* ×15 min), which then kept at −80°C until use. A small portion of the liver sample in each group were removed and fixed with 10% formaldehyde. The remaining livers were cut in pieces and rapidly stocked at −80°C until usage.

### Biochemical Analysis

Serum levels of alanine aminotransferase (ALT), aspartate aminotransferase (AST), total bilirubin (TBIL) and hepatic content of hydroxyproline (Hyp) were measured according to the manufacturer’s guidance. Serum indicators related with liver fibrosis including hyaluronic acid (HA), laminin (LN), collagen type IV (IV-C), and procollagen III N-terminal peptide (PIIINP) were determined on the manufacturers’ protocols as described previously (Wei et al., 2018).

### Determination of Cytokine Levels by ELISA

For the determination of cytokines, liver samples were decomposed in ice-cold RIPA buffer containing protease inhibitors and DNase (0.05 mg/ml). After being incubated on ice for half an hour, the admixture was centrifuged (20,000×*g* for 15 min), at 4°C, and the supernatants were adjusted to equal protein concentrations.

The hepatic and serum levels of TNF-α, IL-6 and IL-1β were then determined following the manufacturer’s protocol.

### Histological Examinations

For histological evaluation, liver tissues were embedded in paraffin, and then cut into 5 μm thick sections and stained with hematoxylin–eosin (H&E). Subsequently, Masson’s trichrome and Sirius red staining was performed according to the standard procedure.

### Immunohistochemistry Staining

In order to determine the hepatic α-SMA expression, paraffin-embedded liver tissues were deparaffined and cut into slices of 5 μm thick. The slices were blocked and incubated with anti-α-SMA antibody (1:100) at 4°C for 12 h. After washed with PBS, the sections were then incubated with a biotinylated secondary antibody (1:1) and an avidin–biotin–peroxidase complex. DAB solution was used for color development. The images were captured and photographed by microscope (3DHISTECH Ltd., Budapest, Hungary).

### Immunofluorescence Staining

The formalin-fixed and de-paraffinized liver sections (5 μm) were incubated with primary antibodies including HMGB1(1:100) or NF-κB (1:50) overnight at 4°C. Subsequently, the sections were then incubated with secondary antibodies at room temperature for 2 h after washing with PBS for three times. Liver sections were incubated with DAPI for 10 min after three PBS washes. Images were captured by an inverted microscope (3DHISTECH Ltd., Budapest, Hungary).

### Quantitative Real-Time PCR

Total hepatic RNA was extract by using TRIzol reagent, which was then reverse transcribed into cDNA following the manufacturer’s guidance. The primers applied in the real-time PCR reactions are shown in **Table 1**. The expressions of mRNA were determined by an ABI prism 7500 Sequence Detection System (Applied Biosystems, USA) and calculated with 2^−ΔΔCT^ method.

**Table 1:** Primer sequences for the PCR reactions.

Target genes		Sequences
β-Actin	Forward	5′-CTATCGGCAATGAGCGGTTCC-3′
	Reverse	5′-TGTGTTGGCATAGAGGTCTTTACG-3′
TIMP-1	Forward	5′-ACAGGTTTCCGGTTCGCCTAC-3′
	Reverse	5′-CTGCAGGCAGTGATGTGCAA -3′
Col1α1	Forward	5′-CGAGTATGGAAGICQAAGG-3′
	Reverse	5′-GCAGTGATAGGTGATGTTCT-3′
TGF-β1	Forward	5′-GCAACAACGCAATCTATGA-3′
	Reverse	5′-CAAGGTAACGCCAGGAAT-3′

### Protein Isolation and Western Blot Analysis

Nuclear and cytoplasmic extracts were isolated from the frozen liver tissues using the methods described previously (Andrews and Faller, 1991). The protein concentrations were quantified by Bradford assay. The Laemmli loading buffer were added to the supernatants, followed by boiling for 4 min, and then subjected to Western blot analysis. After blotting against the primary antibodies at 4°C overnight, the membrane was washed with 0.1% (v/v) Tween-20 in Tris-buffered saline (pH 7.4), and then incubated with the secondary antibodies for another 45 min. The bands were detected using chemiluminescence kits (Applygen Technologies Inc., Beijing, China) and analyzed by using the GelPro32 software program (Media Cybernetics, Marlow, UK).

### Statistical Analysis

The results were presented as the mean ± standard deviation (SD) and analyzed by SPSS 19.0 statistical software. Comparisons were obtained by one-way analysis of variance (ANOVA) followed by Tukey’s multiple-comparison tests. It was considered a statistically significance when *P <*0.05.

## Results

### Effects of ICQA on Liver Injury in CCl_4_-Treated Rats

**Figure 2:**
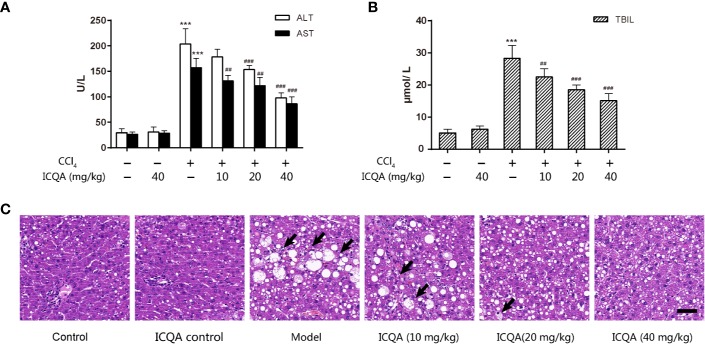
ICQA mitigates CCl_4_-induced liver injury in rats. **(A)** Serum ALT and AST levels; **(B)** serum TBIL level; **(C)** liver sections stained with H&E (Black arrows demonstrating serious hepatic necrosis, ballooning degeneration and inflammatory cell infiltration; scale bar = 50 μm). Data were expressed as mean ± SD. ^***^P < 0.001 vs. control group; ^##^*P <* 0.01, ^###^*P <* 0.001 vs. model group.

In order to observe the protective actions of ICQA on liver fibrosis and clarify the related mechanism, a liver fibrosis model was established in rats by CCl_4_. During our investigation, no death was found in the control and ICQA control group, but two rats were found died in groups of model group and ICQA (10 mg/kg) treated group, respectively. Meanwhile, one death occurred in ICQA (20 and 40 mg/kg) treated group, respectively. Treatment with CCl_4_ dramatically increased the levels of ALT, AST and TBIL (*P <*0.001, **Figures 2A, B**). Further histopathological observation showed that treatment of rats with CCl_4_ for 8 weeks caused serous hepatic necrosis, ballooning degeneration as well as inflammatory cell infiltration. Whereas, ICQA treatment dramatically alleviated the increased serum levels of ALT, AST and TBIL, and the hepatic pathological changes were also significantly improved. Meanwhile, the hepatic histological changes of rats in ICQA treatment groups were significantly ameliorated (**Figure 2C**). In particular, rats in ICQA control group did not exhibit any liver injury, which are similar to the rats in control group.

### Effects of ICQA on Liver Fibrogenesis in CCl_4_-Treated Rats

**Figure 3:**
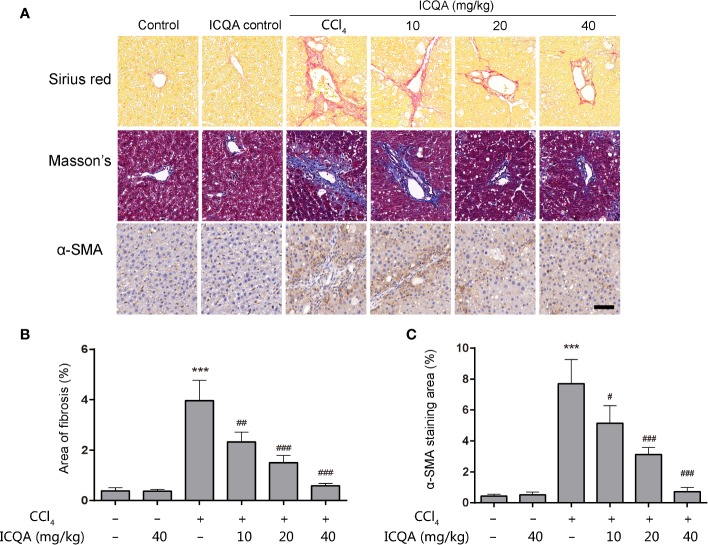
ICQA mitigates CCl_4_ induced liver fibrosis *in vivo*. **(A)** stainings of Sirius red, Masson and α-SMA of liver sections (scale bar = 50 μm); **(B)** the area of fibrosis in liver; **(C)** the area of α-SMA staining in liver. Data were expressed as mean ± SD. ^***^P < 0.001 vs. control group; ^#^*P* < 0.05, ^##^*P* < 0.01, ^###^*P* < 0.001 vs. model group.

As shown in **Table 2**, the contents of the fibrosis indicators were all markedly increased in rats of model group (*P <*0.001). In addition, it was found that the Sirius red and Masson’s staining were significantly enhanced in the livers of rats treated with CCl_4_ (**Figures 3A, B**), indicating the excess collagen deposition. Meanwhile, the hepatic expression of α-SMA was also remarkably upregulated (**Figures 3A, C**). Whereas, ICQA showed dramatically protective effect on liver fibrosis *via* inhibiting the up-regulated expression of hepatic α-SMA and dose-dependently suppressing the hepatic Hyp as well as the serum fibrosis markers (**Table 2** and **Figure 3**). What’s more, the protective actions of ICQA on liver fibrosis were further confirmed by the results of Sirius red and Masson’s staining. Taken together, the above results suggested that ICQA had protective effect on liver fibrosis *in vivo*.

**Table 2:** Effects of ICQA on the serum levels of HA, LN, IV-C, PIIIP and hepatic content of Hyp.

Group	HA (U/L)	LN (ng/ml)	IV-C (ng/ml)	PIIIP (ng/ml)	Hyp (μg/g tissue)
**Control**	28.25 ± 4.17	14.69 ± 2.46	14.88 ± 3.81	2.20 ± 0.29	106.13 ± 11.04
**ICQA control**	28.00 ± 3.46	14.31± 1.13	14.25 ± 1.60	2.33 ± 0.23	105.25 ± 12.09
**Model**	212.00 ± 18.86^***^	107.81 ± 12.43^***^	113.06 ± 12.01^***^	4.96 ± 0.30^***^	205.13 ± 18.53^***^
**ICQA (10 mg/kg)**	180.38 ± 11.64^##^	92.31 ± 8.60^#^	89.75 ± 11.63^##^	3.49 ± 0.39^###^	151.63 ± 13.16^###^
**ICQA (20 mg/kg)**	153.38 ± 14.69^###^	75.31 ± 8.98^###^	70.50 ± 11.99^###^	2.73 ± 0.32^###^	123.50 ± 8.62^###^
**ICQA (40 mg/kg)**	114.88 ± 16.77^###^	57.75 ± 8.89^###^	51.63 ± 8.24^###^	1.99 ± 0.23^###^	97.25 ± 10.63^###^

^***^P < 0.001 vs. control group; ^#^P < 0.05, ^##^P < 0.05, ^###^P < 0.001 vs. model group.

### Effects of ICQA on the Hepatic Expression of TGF-β1, TIMP-1 and COL1α1 in CCl_4_-Treated Rats

**Figure 4:**
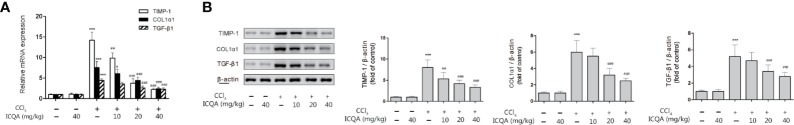
Effects of ICQA on the hepatic expression of TGF-β1, TIMP-1 and COL1α1 in CCl_4_-treated rats. **(A)** mRNA expression; **(B)** protein expression. Data were expressed as mean ± SD. ^***^P <0.001 vs. control group; ^##^*P <*0.01, ^###^*P <*0.001 vs. model group.

TGF-β1 is considered to be one of the most important regulators of HSC activation, and TIMP-1 is critical in the remodeling of matrix. Meanwhile, type I collagen α 1(COL1α1), produced by activated HSCs, is the major ECM component. In order to detect the effect of ICQA on liver fibrosis, the expressions of TGF-β1, TIMP-1 and COL1α1in rat liver were detected in our study. As a result, the expressions of hepatic TGF-β1, TIMP-1 and COL1α1 were all significantly increased in rats treated with CCl_4,_ and administration of ICQA led to a significant reduction. Meanwhile, the expressions were not found to be changed in rats of the ICQA control group (**Figure 4**).

### Effects of ICQA on the Inflammation in CCl_4_-Treated Rats

**Figure 5:**
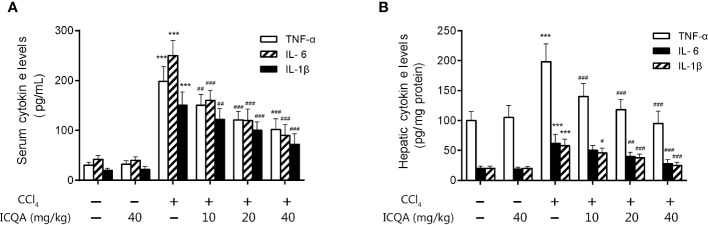
ICQA mitigates inflammation in CCl_4_-treated rats. **(A)** levels of TNF-α, IL-6 and IL-1β in serum; **(B)** hepatic levels of TNF-α, IL-6 and IL-1β. Data were expressed as mean ± SD. ^***^P < 0.001 vs. control group; ^#^*P <* 0.05, ^##^*P <* 0.01, ^###^*P <* 0.001 vs. model group.

Moreover, the action of ICQA on serum and hepatic levels of inflammatory cytokines was assessed in our study. It was found that the contents of TNF-α, IL-6 and IL-1βwere all remarkably increased in the rats treated with CCl_4_ compared with those in control group (*P <*0.001, **Figure 5**). While the expressions of the inflammatory cytokines were decreased dose dependently by ICQA treatment, indicating that the protective action of ICQA on liver fibrosis might be related with the ability to attenuate inflammation.

### Effects of ICQA on HMGB1 Expression and Its Cytoplasmic Translocation in the Liver of Rats Treated With CCl_4_

**Figure 6:**
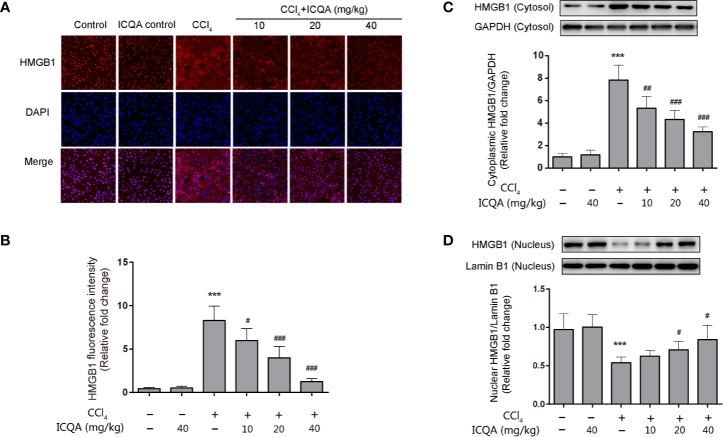
Effects of ICQA on the hepatic expression of HMGB1 and its cytoplasmic translocation in CCl_4_-treated rats. **(A)** fluorescence staining of HMGB1 in the liver (scale bar = 50 μm); **(B)** fluorescence intensity; **(C**, **D)** nuclear and cytoplasmic expressions of HMGB1 in rat liver determined by Western blots. Data were expressed as mean ± SD, ^***^P < 0.001 vs. control group; ^#^*P <* 0.05, ^##^*P <* 0.01, ^###^*P <* 0.001 vs. model group.

HMGB1 translocation and release play a crucial part in liver fibrogenesis. Immunofluorescence staining in our experiment clearly revealed that hepatic HMGB1 was predominantly located in the nucleus of hepatocytes in rats of control and ICQA control group. However, it was found to translocate from the nucleus to the cytoplasm in liver of rats treated with CCl_4_. Meanwhile, the fluorescence intensity of HMGB1 was remarkably decreased in rats in ICQA treated group (**Figures 6A, B**). To further find out whether the distribution of HMGB1 is altered in rats treated with CCl_4_, Western blotting analysis was carried out to observe the distribution of HMGB1 in cytoplasmic and nuclear fraction. As a result, a remarkable increase of the cytoplasmic HMGB1 expression and simultaneously a decrease of nuclear HMGB1 expression were found in rats treated with CCl_4_. Whereas, these changes can be reversed by the treatment of ICQA dose-dependently (**Figures 6C, D**).

### Effects of ICQA on the Hepatic Expression of TLR4 in CCl_4_-Treated Rats

**Figure 7:**
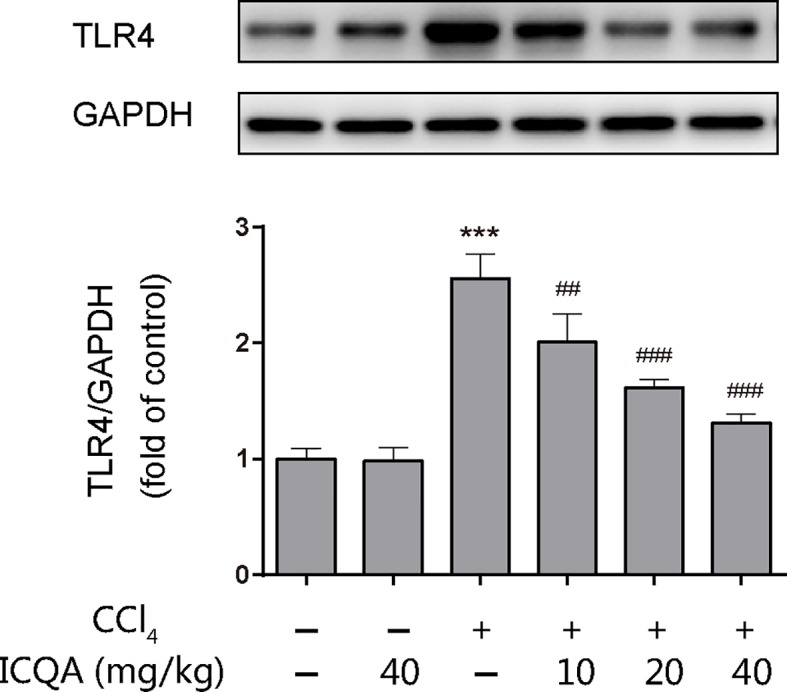
Effects of ICQA on the hepatic expression of TLR4 in CCl_4_-treated rats. Data were expressed as mean ± SD, ^***^P < 0.001 vs. control group; ^##^*P <* 0.01, ^###^*P <* 0.001 vs. model group.

The expression of TLR4 was examined by western blotting analysis and the results showed that treatment of rats with CCl_4_ for 8 weeks upregulated the hepatic protein expression of TLR4. However, ICQA treatment dose-dependently decreased the expression of TLR4 (**Figure 7**). Meanwhile, no alterations were found in the TLR4 expression in rats treated with ICQA alone.

### Effects of ICQA on the Nuclear Translocation of NF-κB in the Liver of CCl_4_-Treated Rats

**Figure 8:**
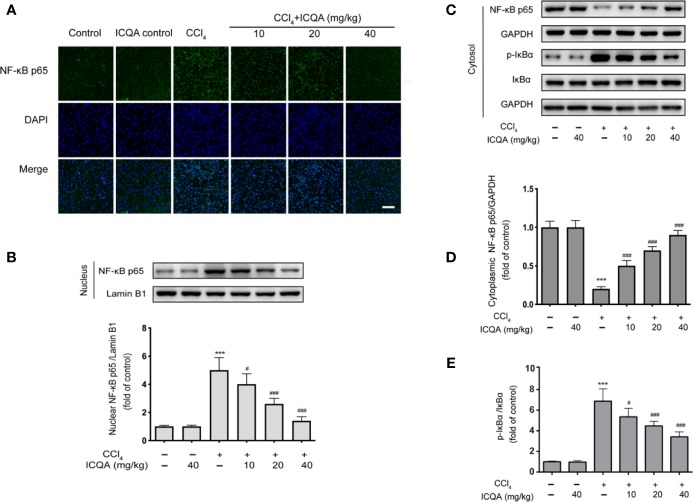
Effects of ICQA on the NF-κB activation in liver of CCl_4_ treated rats. **(A)** Fluorescent micrographs of NF−κB p65-loaded liver section (scale bar = 50 μm); **(B)** protein expression of NF-κB p65 in the nuclear fraction of rat liver; **(C**–**E)** protein expression of NF-κB p65, p-IκBα and IκBα in the cytoplasmic fraction of rat liver. Data were expressed as mean ± SD, ^***^P < 0.001 vs. control group; ^#^*P <* 0.05, ^##^*P <* 0.01, ^###^*P <* 0.001 vs. model group.

According to the results of immunofluorescence staining, the nuclear translocation of NF−κB p65 was found to be dramatically promoted in the liver of rats of model group, which was prevented by the administration of ICQA (**Figure 8A**). To further find out whether the distribution of NF−κB p65 is altered in rats treated with CCl_4_, Western blotting analysis was performed. As a result, a remarkable increase of the NF-κB p65 expression in the nuclear fraction and simultaneously a decrease of NF-κB p65 expression in the cytoplasmic fraction were found in rats treated with CCl_4_. Whereas, these changes can be reversed by the treatment of ICQA dose-dependently (**Figures 8B–D**). In addition, **Figures 8C, E** indicated that the protein expression level of p−IκBα was upregulated in the model group, while ICQA downregulated the p−IκBα level significantly.

## Discussion

Liver fibrosis is critical in the pathogenesis of liver cirrhosis and hepatocellular carcinoma. Unfortunately, no effective therapeutic approaches are available for this disease currently. Thus, it is urgent to explore the mechanisms of liver fibrosis and develop new drugs.

Previous study suggested that polyphenols can suppress inflammation both *in vitro* and *in vivo* (Chen et al., 2018b). In addition, relationship studies focus on structure activity demonstrated that the antioxidative, antitumor, and hepatoprotective activities were associated with the numbers of caffeoyl groups in hydroxycinnamic acids (Xu et al., 2012; Chen et al., 2018a). Particularly, ICQA (dicaffeoylquinic acids) has been proved to have better anti-oxidative activity than chlorogenic acid (caffeoylquinic acids), which possesses significantly hepatoprotective properties, and the protective actions might be owing to its inhibition on TLR4/NF-κB signaling pathway (Xu et al., 2012; Shi et al., 2013; Yuan et al., 2017a). ICQA had been shown to possess protective effect on liver injury induced by hepatitis B virus infection (Hao et al., 2012). However, little is known about its action on liver fibrosis. Moreover, previous study indicated that the bioavailability of ICQA was 22.6%, and a total of 32 metabolites had been determined in rats (Wang et al., 2017). It is well known that the most challenge issue for developing polyphenolics as chemoprevention is the low oral bioavailability (Teng and Chen, 2019). Thus, intervention *in vivo* needs to fully understand how these molecules interact with pathological processes (Teng and Chen, 2019; Wang et al., 2020). Our present study firstly confirmed that ICQA exhibited significant hepatoprotective effect against liver injury induced by CCl_4_ in rats, indicated by dose dependently decreasing the elevated levels of serum parameters, and remarkably improving the histopathological changes. Moreover, our results indicated that administration of ICQA at doses from 10 to 40 mg/kg could attenuate liver fibrosis as demonstrated by the improvement of Sirius red and Masson’s staining. ICQA treatment can also inhibit the expression of COL1α1, which is a well-known markers of HSC activation. In addition, previous investigation confirmed that TGF-β1 is a critical regulator of fibrogenesis in chronic liver diseases (Bissell et al., 2001). Besides, the levels of TIMP-1 seemed to be firmly related with the pathological degree of liver fibrosis in patients, and suppress the expression of TIMP-1 can attenuate the progression of liver fibrosis (Nie et al., 2001; Mannello and Jung, 2008). In our study, it was found that treatment of rats with ICQA could significantly and dose -dependently inhibit the expression of COL1α1, TGF-β1 and TIMP-1 at both mRNA and protein levels, which indicated the potential therapeutic action of ICQA for liver fibrosis.

The mechanism of liver fibrogenesis is complicated. Indeed, numerous scientific publications supported the vital role of inflammation in the pathogenesis of various forms of diseases including liver fibrosis (Czaja, 2014; Pei et al., 2014; Zhang et al., 2016; Wang et al., 2019). Under pathological condition, HMGB1 is passively excreted from damaged cells or actively secreted from the activated immune cells. The HMGB1 released by the proinflammatory stimuli can further increase the production of multiple inflammatory cytokines (Li et al., 2014). In addition, it is also crucial for neutrophil, HSCs recruitment as well as migration (Heymann and Tacke, 2016). All these evidences illustrated that HMGB1 is vital in the progression of liver inflammation and fibrogenesis. In our present study, it was found that the expression of HMGB1 in cytosolic was remarkably upregulated in the liver of rats treated with CCl_4_. The increased HMGB1 levels were considerably restrained by the treatment of ICQA. Further immunofluorescence assay revealed that HMGB1 distributed mainly in the nuclear fraction, with trace detected in the cytoplasmic fraction of hepatocytes in control group. Nevertheless, the cytoplasmic HMGB1 were enhanced by the treatment of CCl_4_. ICQA ameliorated the cytoplasmic translocation of HMGB1 in the liver. Furthermore, the inhibition of cytoplasmic translocation of HMGB1 by ICQA was consistent with its effect on the inhibition of inflammatory cytokines. These results illuminated that the inhibition of cytoplasmic translocation of HMGB1 by ICQA was consistent with its anti-inflammatory and its protective effect against liver fibrosis. However, the mechanisms of the inhibitory effect of HMGB1 cytoplasmic translocation by ICQA require further investigation.

In fact, chronic hepatic inflammation is closely related with fibrosis. Extracellular HMGB1 can interact with TLR4, which is expressed on hepatocytes, cholangiocytes, and HSCs (Li et al., 2014). Ekihiro (Seki et al., 2007) demonstrated that TLR4, but not TLR2, activates myofibroblast and hepatic fibrogenesis. In addition, it was found that the hepatic fibrogenesis was significantly reduced in *Tlr*4-mutant mice. Meanwhile, TLR4 SNP leads to a T399I change caused a remarkably decreased risk for fibrosis development in patients with chronic liver injury (Huang et al., 2007). Therefore, the inhibition of TLR4 seemed be a prospective strategy for the therapy of hepatic fibrosis. Previous evidences suggested that endogenous TLR4 ligands participate in liver fibrosis. For example, MicroRNA-326, a small and endogenous noncoding RNA, was found to attenuate the hepatic stellate cell activation and the liver fibrosis by inhibition of TLR4 signaling pathway (Liao et al., 2019). In addition, Wnt2b, serves as an endogenous inhibitor of TLR4 signaling, exhibited an inhibition on the HSCs activation and mitigated liver fibrosis (Yuan et al., 2017b). Particularly, dietary flavonoids, potential therapeutic agents in the therapy of liver fibrosis, have been proved to possess the ability to inhibit inflammation by modulating intracellular NF-κB signaling through TLR4 (Geng et al., 2017; Chen et al., 2018a). The interaction of HMGB1 to TLR4 activates an inflammatory response and has been indicated to participate in liver fibrosis (Li et al., 2016). After binding, MyD88- dependent/independent signaling cascades, the two crucial intracellular signaling pathways, are activated (Li et al., 2016). The MyD88-dependent pathway signals regulate the expression of proinflammatory cytokines and other immune-related genes (Gao et al., 2012; Xin et al., 2017). Under normal condition, NF−κB combines with IκBα, and inhibited the nuclear translocation of NF−κB p65. Otherwise, IκBα is dissociated, which resulted in the nuclear translation of NF−κB p65 after stimulus. Our study showed that ICQA treatment could dose-dependently decrease the hepatic expression of TLR4 in rats treated with CCl_4_. In addition, our results indicated that ICQA could remarkably reduce the phosphorylation of IκBα and the activation of NF−κB. However, whether other mechanisms are involved in the protective action of ICQA on liver fibrosis need to be further investigation.

## Conclusion

In summary, ICQA has therapeutic potential against liver fibrosis. The mechanism may be related with, at least in part, by inhibiting inflammation through regulating the HMGB1/TLR4/NF-κB signaling pathways.

## Data Availability Statement

All datasets generated for this study are included in the article/supplementary material.

## Ethics Statement

The animal study was reviewed and approved by Institutional Animal Care and Use Committee of the Peking Union Medical Hospital, Chinese Academy of Medical Sciences and Peking Union Medical College.

## Author Contributions

XL, DM, and BZ conceived and designed the experiments. KH and RZ assisted with the experiments. XL wrote the paper. KH, BZ and RZ critically revised the manuscript. All the authors read and reviewed the final manuscript.

## Funding

This work was supported by the CAMS Innovation Fund for Medical Science (CAMS-2017-I2M-1-011).

## Conflict of Interest

The authors declare that the research was conducted in the absence of any commercial or financial relationships that could be construed as a potential conflict of interest.

## Abbreviations

ICQA, Isochlorogenic acid A; CCl_4_, Carbon tetrachloride; HSCs, Hepatic stellate cells; α-SMA, α-smooth muscle actin; HMGB1, High-mobility group box 1; TLR, Toll like receptor; NF-κB, Nuclear factor-κB; ECM, Extracellular matrix; TNF-α, Tumor necrosis factor-α; IL-6, Interleukin-6; IL-1β, Interleukin-1β; TGF-β1, Transforming growth factor-β1; TIMP-1, Tissue inhibitor of metalloproteinase 1; COL1α1, Type I collagen α1; DAMPs, Damage-associated molecular patterns; RAGE, Receptor for advanced glycation end products; ALT, Alanine aminotransferase; AST, Aspartate aminotransferase; TBIL, Total bilirubin; Hyp, Hepatic hydroxyproline; HA, Hyaluronic acid; LN, Laminin; IV-C, Collagen type IV; PIIINP, Procollagen III N-terminal peptide; RIA, Radioimmunoassay; H&E, Hematoxylin–eosin; SD, Standard deviation.
